# Proxy Measures and Novel Strategies for Estimating Nitrogen Utilisation Efficiency in Dairy Cattle

**DOI:** 10.3390/ani11020343

**Published:** 2021-01-29

**Authors:** Anna Lavery, Conrad P. Ferris

**Affiliations:** Agri-Food and Biosciences Institute Hillsborough, Large Park, Co. Down, Hillsborough BT26 6DR, UK; conrad.ferris@afbini.gov.uk

**Keywords:** nitrogen excretion, blood urea, milk urea, mid-infrared spectroscopy, near-infrared spectroscopy

## Abstract

**Simple Summary:**

Dairy cow diets contain nitrogen, mostly in the form of protein. However, dietary nitrogen is used with a low efficiency for milk production, and much of the unused nitrogen is converted to urea and excreted in urine and faeces (manure). Nitrogen within manure can then be lost to the environment, and this is a particular issue when dairy cows are offered diets containing excess dietary protein. As a result, there is increasing pressure on the dairy sector to improve the efficiency with which dairy cows utilise dietary nitrogen. While nitrogen utilisation efficiency can be measured accurately on research farms, this is more difficult on commercial farms. For that reason, there is much interest in developing low-cost and easy-to-use proximate measures that can provide accurate estimates of nitrogen utilisation. This review examines a number of proximate analyses that are already used as indicators of nitrogen use efficiency in dairy cows (e.g., blood urea and milk urea), and a number of more novel measures that may have potential for use in the future (including analysis of milk, blood, urine, breath, and predictions of intake). These ‘proxy’ measurements can be used to improve feeding management and might be used to monitor adherence to legislation.

**Abstract:**

The efficiency with which dairy cows convert dietary nitrogen (N) to milk N is generally low (typically 25%). As a result, much of the N consumed is excreted in manure, from which N can be lost to the environment. Therefore there is increasing pressure to reduce N excretion and improve N use efficiency (NUE) on dairy farms. However, assessing N excretion and NUE on farms is difficult, thus the need to develop proximate measures that can provide accurate estimates of nitrogen utilisation. This review examines a number of these proximate measures. While a strong relationship exists between blood urea N and urinary N excretion, blood sampling is an invasive technique unsuitable for regular herd monitoring. Milk urea N (MUN) can be measured non-invasively, and while strong relationships exist between dietary crude protein and MUN, and MUN and urinary N excretion, the technique has limitations. Direct prediction of NUE using mid-infrared analysis of milk has real potential, while techniques such as near-infrared spectroscopy analysis of faeces and manure have received little attention. Similarly, techniques such as nitrogen isotope analysis, nuclear magnetic resonance spectroscopy of urine, and breath ammonia analysis may all offer potential in the future, but much research is still required.

## 1. Introduction

Dairy cow diets supply nitrogen (N), primarily in the form of protein. In the rumen, dietary protein is degraded into peptides, amino acids, and ammonia (NH_3_), with the latter utilised by rumen bacteria to synthesis microbial protein. It has been estimated that the minimum rumen fluid ammonia concentration for rumen microbial growth is 5.0 mg/100 mL rumen fluid [1] but the minimum rumen ammonia concentration for maximum digestion can vary greatly due to fermentability of feed [2]. Microbial protein and undegraded dietary protein are then digested post-rumen, and the resulting amino acids absorbed and used for maintenance, growth, reproduction, and milk production. However, NH_3_ not utilised by rumen microbes is absorbed through the rumen wall into the blood, where it is detoxified by the liver by conversion to urea, and then excreted primarily in urine. In addition, undigested N is excreted in faeces. However, ruminants have the ability to recycle urea back into the gastrointestinal tract via saliva. In dairy cows, salvia inflow of urea was estimated to be the equivalent of 47 to 61% of total urea inflow into the gut with inflows dependent on dietary N content [3]. Indeed, as dietary N decreases the proportion of urea N recycled into the gastrointestinal tract increases [4]. The recycled urea N can be used as a source of ammonia for microbial protein synthesis.

The term nitrogen use efficiency (NUE) describes the efficiency with which cows utilise dietary N. While the term is not always used consistently, within this paper NUE is used to describe the efficiency with which dairy cows convert dietary N into milk N (i.e., milk N/N intake). While NUE in dairy systems can be extremely variable, ranging from 15 to 40%, NUE is typically low ~25% [5]. This low efficiency is a cause of concern due to the environmental impact of N losses.

Nitrogen losses from manures in the form of NH_3_ can cause terrestrial eutrophication when deposited on sensitive habitats, resulting in biodiversity loses and soil acidification [6]. In addition, NH_3_ reacts with atmospheric acids such as sulphuric and nitric acids to form particles that contribute to fine particulate matter (<2.5 µm) and which are a threat to human health [7]. Manure also contributes to agricultural Nitrous oxide (N_2_O) emissions [8], a potent greenhouse gas with a global warming potential almost 300 times that of carbon dioxide [9]. Furthermore, N losses from manure to watercourses, primarily via leaching, causes eutrophication [10]. In addition, protein is generally the most expensive component of dairy cows’ diets, so inefficient use represents an economic loss. The production of protein feeds in some parts of the world also threaten ecosystems due to deforestation, changes in land-use, and increased resource consumption [11]. As a consequence, legislative, economic, environmental, and societal pressures are focusing attention on both reducing N inputs into, and N losses from dairy systems. 

Historically, in pursuit of higher milk yields, dairy cows in many countries have been offered diets containing excess dietary protein, resulting in low NUE [12]. Low NUE with increasing dietary crude protein (CP) is mainly due to inefficient conversion of degraded dietary protein to microbial protein, especially at times when protein degradation in the rumen is rapid [13]. Indeed, given the strong linear relationship between N intake and N excretion [14,15], reducing dietary protein level is a key tool by which to reduce N excretion and improve NUE [16]. As a result, NUE has become an important performance indicator for dairy farms [17]. 

Assessing NUE and N excretion on farms can be difficult without detailed information on feed intakes and diet composition. Consequently, there is much interest in the development of proximate or ‘proxy’ measurements that are low cost, easily implemented on farms, and which provide accurate estimates of NUE, so as to facilitate improved nutritional management, to inform policy, and to provide tools which can be used to monitor adherence to legislative requirements. In addition, alternative strategies for examining NUE within research settings are also of interest. This review examines a number of proximate analyses that can be used as indicators of NUE in dairy cows, including blood urea nitrogen (BUN) and milk urea nitrogen (MUN), together with a number of other more novel measures.

## 2. Strategies for Estimating the Efficiency of Nitrogen Utilisation

### 2.1. Blood Urea Nitrogen (BUN)

Ammonia not utilised by rumen bacteria is absorbed from the rumen into the blood, before being detoxified in the liver to urea and eventually excreted, primarily via the kidneys in urine. As urea readily diffuses in and out of blood cells, BUN can also be referred to as plasma urea N (PUN), and in the literature, both are considered to be the equivalent of each other [18]. While the majority of urea in blood is excreted in urine, blood urea can also be recycled into the rumen or transferred into the gastrointestinal tract. This is particularly applicable when N intakes are low, as renal urea clearance rate decreases and renal urea reabsorption increases, as the need to retain N increases. Therefore kidney function is central to the relationship between BUN and urinary N excretion [19]. 

Given that blood urea N levels can fluctuate throughout the day, with the highest levels normally detected 4 to 6 h after feeding, the time of sampling can influence BUN concentrations. However, this pattern is influenced by dietary CP: for example, Kauffman and St. Pierre [20] reported PUN to be unaffected by the time of sampling when cows were offered a diet with a content of 13% but a downward trend in PUN concentrations over time when offered a 17% CP diet. This may reflect increased urea clearance from blood with higher CP levels in the diet. Fluctuations in BUN prior to and following a meal may also be influenced by the Labile N pool [21]. Labile N is primarily found as urea in the blood plasma, but also in organs with a high protein turnover rate, such as the liver, kidneys, and intestine. These ‘protein’ pools are then available when protein is low, for example, prior to feeding. Therefore peaks in BUN following a meal may be lower than expected due to the buffering effect of the protein absorbed and stored in these tissues [21]. Blood urea N levels are also sensitive to water intake, with BUN concentrations in dairy cows observed to increase from 8.5 mg/dL to 15.6 mg/dL with a 50% reduction in water intake [22], as a consequence of low water intakes reducing urine production. Thus season can impact BUN levels as it has been estimated that each one degree Celsius increase in ambient temperature can increase water intake by 1.5 kg/day [23]. In addition to ambient temperature, the water intake of a dairy cow can be influenced by factors such as milk production, sodium intake, and body weight [23]. Despite diurnal variations, BUN is routinely measured on some farms as part of a herd metabolic profile to identify nutritional constraints before they impair herd performance [24]. A plasma urea concentration >1.7 mmol/L was identified as satisfactory for dairy cows under United Kingdom (UK) conditions [25]. In an analysis of data from 35,000 dairy cows in the UK, 16% of cows in early lactation had plasma urea concentrations <1.7 mmol/L, suggesting the ration either to be deficient in effective rumen degradable protein (ERDP), or to be adequate in ERDP but the cows having low intakes [24]. While 21.9% of cows in this same study had a ‘high’ plasma urea concentration (>3 mmol/L), the authors highlighted this due to potential concerns about cow fertility, rather than concerns about cows being offered diets with increased environmental risk [24].

**Figure 1:**
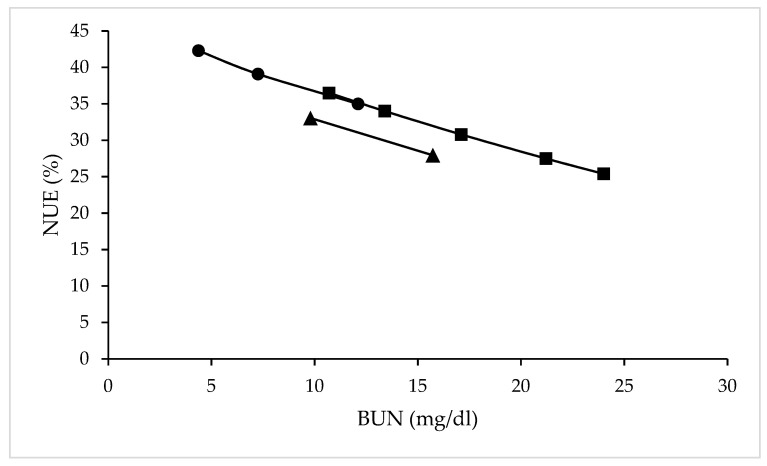
Relationship between blood urea nitrogen (BUN) concentration (mg/dL) and nitrogen use efficiency (NUE, %) from studies of (▲) Kauffman and St-Pierre (2001), (■) Colmenero and Broderick (2006) and (●) Law et al. (2009).

Blood urea N levels are often measured as an indicator of protein sufficiency. For example, a meta-analysis of 15 studies involving beef and dairy cattle published between 1982 and 2002 reported an average BUN of 12 mg/dL at N intake of 203 g/day, while BUN values ranged from 4 to 25 mg/dL and N intakes from 49 to 729 g/day [19]. Colmenero and Broderick (2006) [16] reported BUN levels of 10.7, 13.4, 17.1, 21.2 and 24.0 mg/dL in dairy cows, corresponding to dietary protein levels of 13.5, 15.0, 16.5, 17.9 and 19.4%, respectively, while NUE decreased linearly from 36.5% with the 13.5% CP diet, to 25.4% with the 19.5% CP diet (Figure 1). Furthermore, Kauffman and St-Pierre (2001) [20] found dietary CP levels of 13 and 17% resulted in average BUN concentrations of 9.8 and 15.7 mg/dL, respectively, with average NUE of 33.1 and 28.0% respectively (Figure 1). In one of the few long-term studies examining the impact of diet protein levels in dairy cow performance, plasma urea levels were 1.56, 2.59, and 4.32 mmol/L for cows offered diets containing 11.4, 14.4, and 17.3% CP over the first 150 days of lactation [26]. While this study highlighted the strong relationships between diet CP, BUN, and NUE (Figure 1), milk yield was reduced with the two lower protein diets compared to the high protein diet (25.4, 31.8, and 35.4 kg/day, for the 11.4, 14.4, and 17.3% diet treatments), even though BUN with the 14.4% CP treatment was considerably higher than the 1.7 mmol/L cited by Whitaker (2004) [25] as optimum. 

With regards to the relationship between BUN and urinary N excretion, Kohn et al. (2005) [19] found urinary N can be predicted as clearance rate of kidneys (L of blood cleared of urea per day) × body weight x BUN, with the authors suggesting a standard clearance rate of 1.3 L/day for cattle. Similarly, Kume et al. (2008) [27] observed a positive relationship between PUN and urinary N excretion. However, in the latter study, kidney clearance rates differed between dry cows and lactating cows, 1.33 and 2.08 L/day, respectively. In addition, Burgos et al. (2007) [28] found a strong relationship between PUN levels and urinary urea N excretion (R^2^ = 0.94). Nevertheless, the invasive nature of blood sampling limits the role of BUN as a proximate measure of NUE on a regular basis at a whole herd level. In view of this, the potential of mid-infrared spectroscopy (MIRS) analysis of milk to predict blood metabolites, including BUN has been examined. Indeed, Luke et al. (2019) [29] reported a coefficient of determination of BUN of 0.90, while Benedet et al. (2019) [30], found that BUN could be predicted with a coefficient of determination of 0.58. The lower figure for the latter study may reflect the more limited range of BUN levels in that study, highlighting the need for robust calibration sets to enable accurate predictions. 

Given that urea readily diffuses across cellular membranes and establishes an equilibrium with other body fluids, it is unsurprising that there is a strong relationship (R^2^ = 0.84) between BUN and MUN in dairy cows [18], with this the basis for utilising MUN to predict N excretion and NUE in dairy cows [18,20,31,32]. 

### 2.2. Milk Urea Nitrogen (MUN)

Milk urea N (MUN) is commonly used as a proximate measure of N utilisation of dairy cows [33], and in contrast to BUN, samples are easily obtained non–invasively. Factors influencing MUN concentrations, and its relationship with urinary urea excretion, have been extensively reviewed by Spek et al. (2013) [21] and Gulinski et al. (2016) [34], thus this section will only highlight some key issues. 

Many studies have examined relationships between dietary protein levels and MUN concentrations in dairy cows. For example, in a meta-analysis of 50 trials conducted in Finland and Sweden with grass or grass-legume silage based diets, mean dietary CP was 16% (ranging from 11.1 to 24.9%), while average MUN was 13.3 mg/dL, ranging from 3.8 to 27.0 mg/dL [32]. In this analysis, each percentage change in dietary CP, increased MUN by 1.7 mg/dL. Similarly, Aguilar et al. (2012) [35] found that each percentage change in dietary CP concentration between 13.6 to 19.9% resulted in a 1.04 and 1.24 mg/dL increase in MUN, at milk yields of 43 and 30 kg/day, respectively. Thus in general, each unit change in dietary CP could be expected to increase MUN by 1 to 2 mg/dL. 

Relationships between MUN and urinary N excretion, and NUE, have also been examined using grazing dairy cows. With grazing dairy cows, when pasture CP content increased from 18.4% to 22.6%, average MUN increased from 11.1 to 15.8 mg/dL, respectively, with a linear relationship between MUN concentration and N intake. This was associated with an increase in urinary N of 17.8 g/day per mg/dL increase in MUN [36]. In the meta-analysis of 50 studies undertaken by Nousiainen et al. (2004) [32], urinary N excretion increased 13.4 g/day per 1 mg/dL increase in MUN (R^2^ = 0.735). In the same study, the efficiency of N utilisation for milk protein synthesis decreased by an average of 6.6 g/kg, per unit increase in MUN concentration (R^2^ = 0.567). More recently, Huhtanen et al. (2015) [12] reported that urinary N excretion increased by 5.8 g/day per 1 mg/dL increase in MUN concentration, while NUE decreased by 1.57 g/kg (milk N/N intake, g/kg) per 1 mg/dL increase in MUN concentration. The variable nature of the responses were further highlighted using a number of published equations, with a MUN of 10 mg/dL resulting in a predicted urinary N excretion of between 128 to 223 g/day [21]. When MUN was restricted to between 5–15 mg/dL, the prediction accuracy of MUN was low (R^2^ = 0.23) [21]. 

Part of the variability in responses can be attributed to renal reabsorption of urea. At MUN concentrations above 25 mg/dL, observed at a dietary CP of 21%, the relationship between MUN and urinary N excretion was no longer linear [28], although there was a return to a linear relationship (R^2^ = 0.96) when levels above 25 mg/dL were removed from the dataset. Similarly, in a meta-analysis of 23 trials, a linear plateauing relationship was reported between dietary CP concentration and urinary urea N: MUN ratio [21], with the author suggesting that at a dietary CP <17%, renal re-absorption of urea may increase, whereas >17% renal re-absorption of urea remains unchanged. However, a limitation of the current literature is that the majority of studies involve diets with either adequate to an excessive amount of dietary CP, with relatively few studies examining the relationship between MUN and urinary excretion at low dietary CP levels. 

High urinary N: MUN ratios can still be expected with low MUN and dietary CP intake due to the contribution of non-urea N components. In a meta-analysis of 20 trials, Spek et al. (2013) [21] found that non-urea urinary N: MUN ratio is negatively related MUN. The main non-urea components of cattle urine are allantoin (2.2 to 14.2%), creatinine (1.8 to 5.5%), creatine (1.3 to 4.1%), hippuric acid (3.4 to 8.0%), and ammonia (0.3 to 9.1%) and the proportion of these components contributing to the total N in urine is largely influenced by diet, as extensively reviewed by Dijkstra et al. (2013) [37]. For instance, an increase in dry matter intake (DMI) can increase microbial protein synthesis and the excretion of allantoin and hippuric acids, increasing the non-urea urinary N excretion. Although the contribution of the non-urea components may be small compared to urea, which typically contributes 52 to 93% of total N in urine, [37], it should be taken into consideration when estimating urinary urea N excretion based upon predictions between the ratio of urinary N and MUN. 

In addition to dietary CP levels, the type of dietary carbohydrate may also influence the urinary N: MUN ratio. Feeding readily fermentable energy sources can lower concentrations of rumen ammonia, and consequently lower BUN and MUN compared with NDF-rich diets [38]. Indeed Hof et al. (1997) [33] suggest that 80% of the variation in MUN can be attributed to differences in rumen fermentation. Moreover, Cheng et al. (2014) [39] reported no relationship between MUN and NUE, due to the absences of differences in rumen fermentation. In addition, products of fermentation such as short-chain fatty acids, carbon dioxide, NH_3,_ and rumen pH are all factors which influence the transport of ammonia and urea across the rumen wall [40] and are therefore likely to influence the dynamics of BUN concentration, and as a result the relationship between MUN and urinary urea N excretion. 

In addition to diet, a number of other factors influence MUN levels, including analytical techniques [41,42,43]. For example, while MUN was traditionally analysed using ‘wet’ chemistry approaches, it is now routinely predicted using MIRS, with low concentrations of MUN creating challenges. This was highlighted when samples with enzymatically determined MUN values less than 3 mg/dL were analysed using MIRS, with MUN values predicted to be zero, indicating a higher lower detection limit for MUN by infrared spectrometry [21]. Nevertheless, the use of MIRS to predict MUN offers a quick and cost-effective analysis approach for the analysis of large numbers of ‘industry’ samples, although there is still scope to improve accuracy. 

‘Cow’ factors, including cow body weight (BW) and genotype, may also affect MUN. The effect of BW on MUN is likely a consequence of a larger animal having a larger blood urea pool, thus requiring more urea to be excreted in urine [21] to reduce BUN. Indeed, Kauffman and St-Pierre (2001) [20] reported the accuracy of predicting urinary N excretion using MUN to be improved by the addition of BW (R^2^ = 0.98) to the equation (Urinary N (g/day) = 0.0259 × BW (kg) × MUN (mg/dL)). Although regression coefficients reported in the literature, do vary, it is widely accepted that BW influences the relationship between urinary N excretion and MUN. 

While differences in MUN between different breeds of dairy cattle may be partially explained by differences in BW, there are also genetic differences. The heritability of MUN has been investigated in several studies [44,45,46,47,48], with values ranging from 0.13 to 0.59, highlighting that it should possible to breed cattle with lower MUN concentration. However, it is unclear whether selection for lower MUN necessarily results in a concurrent reduction in urinary N excretion and an improvement in NUE. For example, Lopez-Villalobos et al. (2018) [47] reported no significant genetic relationship between MUN and NUE, while Miglior et al. [49] reported a positive relationship between MUN and percentage milk protein. In contrast, a negative relationship between MUN and protein-use efficiency was observed in a meta-analysis [50], while a negative genetic correlation between MUN and the percentage of true protein in milk was found across breeds in a New Zealand study [48]. While inconsistent outcomes may be attributed in part to genetically divergent populations between studies, in general, the literature indicates that selecting for MUN may result in differential portioning of N to body pools, an area that requires further research. 

Thus the cow herself is an important source of variation in MUN [35], with cow variance in MUN estimated to be 4.1 ± 1.1 mg/dL [51,52]. In view of this, individual cow measures of MUN may not be sufficiently reliable to rank cows for NUE. To better account for cow variation (body weight, genetics, breed) it may be more appropriate to establish a within-herd baseline to better evaluate feeding and management practices employed on each farm. Consequently, MUN may be more suited as an indicator of overall diet adequacy within a particular herd [53], rather than as an individual cow management tool. 

While the use of MUN as an accurate predictor of urinary N excretion, and a biomarker of NUE has a number of limitations, as discussed, ‘target’ MUN levels have been developed. For example, with grass-silage based diets, a MUN value of 11.7 mg/dL was suggested consistent with N requirements of rumen microbes being met, while a MUN level above 16 mg/dL, was associated with a reduction in NUE, despite an increase in milk protein yield [32]. Similarly, earlier research [54], based on predicted urinary N excretion of cows fed according to recommendations identified target MUN concentrations of between 10–16 mg/dL, depending on levels of milk production. In addition, further studies that integrated body weight into prediction models [20,31] estimate target MUN concentration of between 8–12 mg/dL for the majority of dairy farms [55], and indeed a target MUN of between 8 to 14 mg/dL has been widely adopted in the industry as indicating a diet which is ‘optimal’ [56]. Nevertheless, there is agreement that the use of MUN as a biomarker to predict NUE and urinary N excretion is most accurate when applied to similar nutritional circumstances to which the models were developed [32].

As a general management tool, milk bulk tank measurement may be a more useful diagnostic tool to evaluate the efficiency of N utilisation on-farm [20,31]. This highlights the need to identify other potential biomarkers of NUE in individual cows. 

### 2.3. Direct Prediction of NUE Using Mid-Infrared Spectroscopy (MIRS) Analysis of Milk

The use of MIRS analysis of milk to predict NUE in early lactation dairy cows was examined by Grelet et al. 2020 [57]. While milk MIRS alone, as a predictor of NUE, had a cross-validation R^2^ of 0.63 and relative error of 17%, the model was improved by the inclusion of parity and milk yield (R^2^ = 0.74, relative error = 14%). Based on the latter, the authors suggested the model should be able to differentiate between cows with low and high NUE. However, while the model was able to predict NUE of cows offered similar diets to those used in the original dataset with similar accuracy (R^2^ = 0.68, relative error = 14%), the accuracy was variable (R^2^ range 0.06 to 0.68, relative error 12 to 34%) when used to predict NUE of cows offered different diets. These findings highlight the need for robust calibration sets encompassing a diverse range of diet types, in order to improve the accuracy and quality of predictions. Nevertheless, these early findings [57] suggest that developments in MIRS analysis of milk may eventually offer a quick and cost-effective predictor of NUE in dairy herds, and this low-cost approach requires further research. 

### 2.4. Near-Infrared Spectroscopy (NIRS) of Faeces 

Near-infrared spectroscopy (NIRS) has been used to predict N fractions within dairy cow faeces [58], with a strong correlation between ‘wet’ chemistry analysis and NIRS predictions for total N, ammoniacal-N, water-soluble N, and undigested dietary N (R^2^ of 0.97, 0.92, 0.91, and 0.90, respectively). However, microbial N from both the rumen and hindgut constitutes a large part of faecal metabolic N [59], and it is more difficult to predict bacterial and endogenous N with NIRS, with a lower R^2^ of 0.78 and a greater standard error of calibration to standard deviation ratio (0.50) [58]. The potential of faeces analysis to predict NUE may be limited by the fact that a large proportion of N excreted by dairy cows is excreted in urine, although lowering dietary CP, results in faecal N representing the larger proportion of excreted N [37]. Nevertheless, to our knowledge, the NIRS analysis of faeces as a predictor of NUE has not been examined. In contrast, manure represents a mixture of both faeces and urine in the proportions excreted by cows, with many studies having examined the use of NIRS of manure to determine manure nutrient content [60,61]. However, as with faeces, we are not aware of any research that has attempted to predict NUE from NIRS analysis of manure. 

### 2.5. Nuclear Magnetic Resonance (NMR) Spectroscopy of Urine

Nuclear magnetic resonance (NMR) is an analytical chemistry tool which can be used to detect multiple metabolites in a sample simultaneously. For example, NMR analysis of the metabolite profile of bovine urine has been used to distinguish between different beef production systems, and to discriminate feeding practises [62]. Consequently, it is expected that the metabolite profile of cows’ urine would differ according to dietary N intake. Indeed, Bertram et al. (2011) [63] examined the metabolite profile of dairy cow urine as a potential biomarker of NUE, and found a high correlation with CP intake (R = 0.80) and NUE (R = 0.74). Furthermore, NMR signals for urea, hippurate, phenylacetylglutamine, and p-cresol sulphate were correlated with and contributed to the prediction of N intake and NUE. A strong signal of urea is to be expected as the major end product of protein metabolism, while hippuric acid, derived from plant phenolic compounds, increased with increasing CP intake. The authors suggested that both phenylacetylglutamine and p-cresol sulphate may be derived from the metabolism of tyrosine residues in proteins. While these findings are promising, to have practical application as a biomarker for NUE further research is needed to determine if relationships are repeatable across a diverse range of diets. Nevertheless, this approach may offer the potential to help develop a better understanding of N utilisation within research settings. 

### 2.6. Nitrogen Isotope Analysis of Plasma, Milk and Hair

Stable isotope fractionation, and in particular N isotope analysis, is ordinarily used to indicate the trophic level of organisms. Fractionation of isotopes is a result of changes in the ratio of heavy to light isotopes. In the case of N, nitrogen-14 (^14^N) and nitrogen-15 (^15^N) have the same number of protons but a different number of neutrons, with the latter (heavier) isotope requiring more energy to break its bonds, while having a slower reaction rate [64]. Therefore some biological pathways discriminate between this difference in mass between ^14^N and ^15^N. This results in differential enrichment of ^15^N, often reported as delta units (δ^15^N; ‰), namely the ratio of ^15^N/^14^N in the sample relative to ^15^N/ ^14^N ratio in the standard. Analysis is conducted using an isotope ratio mass spectrometer. Due to differences in N partitioning in animals, faeces, and milk are generally enriched with and urine depleted in ^15^N relative to the diet [65,66].

During the last decade, a number of studies examined the use of ^15^N isotope as a biomarker of NUE in ruminants [39,64,65,67,68]. There is evidence which suggests N isotope fractionation can predict NUE of dairy cattle at pasture, with a strong negative relationship between the differential fractionation of milk ^1^ δ^15^N − feed δ^15^N and NUE (r^2^ = 0.83), and plasma δ^15^N –feed δ^15^N and NUE (r^2^ = 0.85) [69]. In agreement with these findings, Wheadon et al. (2014) [64] also reported a negative relationship between NUE and plasma ∆^15^N (plasma δ^15^N − diet δ^15^N; r^2^ = 0.45) when dairy cows received zero-grazed fresh herbage. However, milk ^15^N may not be as reliable a predictor of NUE for dairy cows fed pasture high in RDP, as NUE was reduced when there was excess RDP in the diet, resulting in low ^15^N enrichment of milk [64]. More recently, Cantalapiedra-Hijar et al. (2017) [68] used a meta-analysis to show that ∆^15^N animal-diet (the δ^15^N difference between an individual and its diet, ∆^15^N = δ^15^N animal − δ^15^N diet), can predict NUE variation across diets and between individual ruminants reared under similar conditions. The ∆^15^N animal-diet was shown to be negatively correlated with NUE in ruminants reared under different conditions when using treatment means and when using individual animal values (−0.055 and −0.050 g/g, respectively). ^15^N isotope biomarker also has the ability to distinguish individuals in a group, with a significant negative correlation between ∆^15^N animal-diet and NUE at the level of between-animal variation when adjusted for study period and diet (slope of −0.035 g/g) [68]. Unsurprisingly, the most important variables related to N utilisation explaining the link between ∆^15^N animal-diet and NUE were reportedly N metabolism and to a lesser extent rumen fermentation and digestion [68]. Recently, the relationship between urinary N excretion and both ∆^15^N animal-diet in plasma and MUN was described by Nasrollahi et al. (2019) [70]. In addition to both biomarkers being able to independently predict urinary N excretion (∆^15^N animal-diet in plasma, r^2^ = 0.47; MUN, r^2^ = 0.50), the combination of biomarkers in the model, strengthened the prediction ability of the model. This finding indicates the potential relationship between ∆^15^N animal-diet and MUN as a novel predictor of NUE in dairy cows. 

In contrast, Cheng et al. (2011) [65] were unable to confirm a relationship between N isotope fractionation and NUE, although this may have been due to the impact of study design (short-term change-over), dietary ammonia-N content, and mobilisation of body reserves, all influencing ^15^N fractionation [65]. Moreover, absence of a relationship between plasma ^15^N and NUE was attributed to the fact that N isotope fractionation may have been unable to detect subtle differences (<0.3‰) in ^15^N [39]. In addition, Herremans et al. (2020) [71], suggested ∆^15^N may not be reflective of overall NUE but rather an indicator of a shift in N partitioning at a metabolic level. Given the slow turnover rate of ^15^N, a benefit of ∆^15^N animal-diet is the lack of diurnal variation regardless of feeding time. In contrast, the period of time between dietary changes and blood/milk sampling can affect predicting NUE from ∆^15^N animal-diet [68]. Since ^15^N takes time to reach isotopic equilibrium, the proposed time-lag for its use in plasma of ruminants is 45 days [67]. The use of ^15^N isotope fractionation as an indicator of NUE in ruminants is a relatively new concept and as such presents opportunities for further research. Currently, it is not understood how ^15^N enrichment is affected by mobilisation of body reserves, and this could affect estimates of NUE in early lactation dairy cows. 

As N loss in hair and skin cells represents a very small proportion of N losses compared to urine and faeces, little attention has been given to N in hair. Nonetheless, as hair is progressively laid down over time and remains unchanged, isotope signatures and information regarding diet and growth are ‘preserved’. Sequential isotopic analysis of short sections (10 mm) of cattle tail switch hair have been shown to successfully reflect diet compositions and farm management systems over a period of time [72]. For example, while Schwertl et al. (2005) [72] found that cattle hair ^15^N was correlated with animals stocking rate (r^2^ = 0.55), high stocking rates were reflective of high N inputs on farms resulting in a close correlation between ^15^N hair signatures of cow and N input-surplus on farm (r^2^ = 0.78). Feed ^15^N varied greatly between different feeds analysed on farms but this result was consistent with a causal relationship with N input surplus and intensity of farm production system. Correspondingly, Sponheimer et al. (2003) [73] showed that for cattle in similar physical condition, the diet-hair enrichment of ^15^N was 2.3% greater when a high protein diet (19%) was fed compared to a low-protein diet (9%). It is known that with excess protein in the diet, the excretion of ^15^N depleted urea increases [74], and therefore ^15^N diet-tissue fractionations should be higher than for animals on high protein diets. However, it is important to note the period of hair sampling relative to dietary changes can influence findings as evidenced by Sponheimer et al. (2003) [73]. Diet–hair ^15^N of horses varied greatly from 2.5% to 7.8% within the first week following dietary changes but was equilibrated in less than 24 weeks, with less than 1.5% change in ^15^N from week 8 to 24. Thus changes in dietary N content can be detected in N isotope analysis of hair and therefore stable isotope analysis of cattle hair may reflect changes in NUE over time. While further research in this area is needed, this analysis may offer a non-invasive ‘fingerprint’ of NUE of an animal over-time. However, specialised analytical techniques and equipment mean stable isotope analysis is expensive, and although presenting interesting research opportunities, may not be practical for wide-scale application. 

### 2.7. Breath Ammonia 

Although excess rumen ammonia is mostly converted to urea in the liver, a small proportion of ammonia circulating in the blood can diffuse into the lungs and be exhaled in breath. The majority of work in this area has been conducted in human studies, with the levels of BUN and breath ammonia (BA) highly correlated (R = 0.77 − 0.84), with real-time measurements allowing this to be used as a diagnostic tool [75]. However, we are aware of only one study in the literature in which BA levels were investigated as a novel indicator of N metabolism in ruminants. Breath ammonia of heifers offered diets differing in CP content (9, 12, 15, or 18% CP) was assessed using closed-circuit respiratory systems with a facial mask [76]. Breath ammonia increased with increasing dietary CP level and was highly correlated with serum urea N (r = 0.67) and urinary urea N (r = 0.55). However, time and duration of sampling are important with peak BA measured 6 h after the peak serum urea N content. Furthermore, while very low dietary CP (9%) levels are impractical for dairy cows, this diet resulted in a negative BA excretion, which the authors indicated may be due to a lower BA compared to the concentration in the atmosphere. It is also possible that rumen ammonia contributes to BA, although Dewhurst et al. (2001) [77] reported a low correlation between eructated ammonia from rumen headspace gas and ammonia concentration in rumen liquor. Nevertheless, rumen pH in this study was relatively stable (6.37 to 6.77), with the authors concluding that rumen pH is likely a major factor influencing eructated ammonia rather than rumen ammonia concentration per se. While there is limited literature in this area, BA may offer the opportunity of a non-invasive measure of N utilisation in ruminants, and one which is not limited to lactating animals. Future developments in precision livestock farming techniques may make it possible to incorporate BA sensors into automatic milk systems or automatic concentrate feeders. 

### 2.8. By Predicting N Intakes

Automated systems on many research farms allow intakes of individual dairy cows to be recorded with a high degree of accuracy. Provided the N content of all ration components and the N content of milk is recorded regularly, NUE can be determined. For example, a recent meta-analysis of studies indicated that on average approximately 27% of total N consumed by dairy cows is secreted in milk [78]. Measuring intakes of individual cows during grazing is more problematic, as DMI is influenced by climatic conditions, herbage allowance, and daily changes in forage DM, CP, and fibre content. Nevertheless, techniques such as the n-alkane method allows individual cow intakes to be estimated with a good degree of accuracy, thus allowing NUE to be determined, even when cows are grazing. Furthermore, a more complete picture of N utilisation can be obtained by the gold standard approach of ‘whole animal N balance studies’, involving total collection of faeces and urine, as extensively reviewed by Hristov et al. (2019) [79]. However, these techniques are complex, expensive, and labour-intensive and are not applicable for use on commercial farms. 

**Figure 2:**
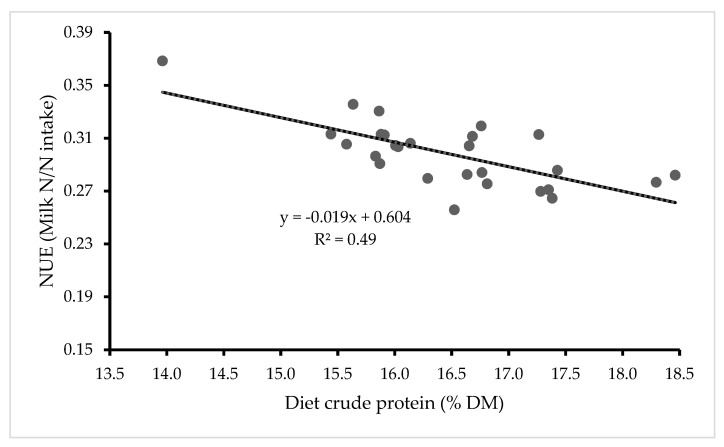
Relationship between NUE and diet protein level over the winter on 27 Northern Ireland dairy farms, based upon predicted dry matter intakes for individual cows (Craig, unpublished data).

Nevertheless, if DMI can be measured or predicted with reasonable accuracy on farms, and detailed information on the N content of rations is available, then NUE can be estimated. For example, the use of complete diet mixer wagons which allow quantities of food dispensed to individual groups of cows to be recorded can facilitate calculation of NUE on a group basis, provided a reasonable estimate of uneaten food is available. When information on quantities of food offered is not available, prediction equations can be used to predict intakes, either at an individual cow level or at a group/herd level. For example, based on monthly predictions of daily DMI using a model specifically for feed- to yield systems [80], and monthly determination of the N content of feeds, a mean NUE of 30% was determined for 27 Northern Ireland farms offering concentrates on a feed-to-yield basis during the winter (Craig, unpublished), and in this study, NUE increased as diet CP decreased (R^2^ = 0.49) (Figure 2).

A number of other approaches exist by which to predict DMI. For example, MIRS, which is routinely used in the dairy industry to predict fat, protein, and lactose content of milk, was recently been used to predict DMI of dairy cows in a number of studies [81,82]. More recently, Lahart et al. (2019) [83] found that MIRS analysis of milk alone was a poor predictor of DMI of grazing lactating dairy cows (R^2^ = 0.30), but when combined with milk yield, milk composition (fat and protein %), cow body-weight, stage of lactation and parity, prediction accuracy improved (R^2^ = 0.64). In addition, Klaffenblock et al. (2017) [84] used MIRS to estimate the different components of dairy cow rations. The R^2^ obtained for feedstuffs (as kg DMI or as % of the ration, respectively) such as pasture (0.63, 0.66), grass silage (0.32, 0.43), maize silage (0.32, 0.33) and concentrates (0.39, 0.34), are promising. In addition, this study demonstrates that MIRS of milk has the potential to provide information on both the N content of milk and dietary N intake, the two parts of the NUE ratio.

Furthermore, while near-infrared spectroscopy (NIRS) analysis is already used as a quick, low cost and accurate way to determine the nutrient content of feeds, its potential to predict DMI and ration digestibility of dairy cows has been investigated, with promising results. Indeed, Garnsworthy and Unal (2004) [85] reported a strong relationship when DMI was predicted directly from NIRS (R^2^ = 0.97), with estimates similar to those derived from the n-alkane technique. Similarly, Decruyenaere et al. (2012) [86] found that grass DMI (r = 0.63–0.88) predicted using NIRS analysis of faeces were correlated with other predictive methods, such as ratio technique and animals performance methods. In contrast, Lahart et al. (2019) [83] reported that NIRS analysis of faeces alone was a poor predictor of DMI (R^2^ = 0.16), with prediction equation accuracy improved when, MIRS analysis of milk, milk yield, milk composition (fat and protein (%)), cow body weight, stage of lactation and parity were included (R^2^ = 0.68). However, the potential of the NIRS analysis of faeces to predict DMI accurately will be dependent on developing robust calibration datasets which encompass the variability in faecal compositions associated with differences in diet composition and DMI [87].

Similarly, a range of sensors with the potential to predict individual cow intakes are currently being developed, and these, if combined with accurate composition of rations offered and milk N content, may allow NUE to be estimated. These include neck-mounted accelerometers, which have been used to determine grazing time and speed of bite, and whilst there are promising developments for the use of these sensors to estimate DMI of grazing ruminants [88,89], further work is needed to improve accuracy due to varying quality and compositions of pasture. Nevertheless, these approaches do require the N content of all diet components to be recorded accurately.

## 3. Conclusions

While BUN is strongly correlated with NUE, the invasive nature of blood sampling poses a limitation for widespread adoption on farms. In contrast, MUN can be sampled non-invasively, and easily measured using MIRS, making it a widely used proximate measure of NUE on farms. Extensive research has demonstrated strong relationships between MUN and N intake, allowing the use of MUN as a herd level predictor of NUE. However, MUN has a limitation as a predictor at an individual cow level. Direct prediction of NUE using MIRS may have potential to improve individual cow predictions, although further research to develop improved calibrations across a wide range of diet types is required. Combining DMI predictions, either at a cow or group level, with detailed information on feed composition, offers another approach to examine NUE, and one which could be more widely adopted if the correct feed composition information was available. At present, other novel techniques such as nitrogen isotope analysis, nuclear magnetic resonance spectroscopy, and breath ammonia analysis offer opportunities to further develop understanding of N utilisation within a research setting but have limited farm application at present. Currently, MUN offers the most practical proximate measure of N utilisation of dairy cows’ on-farm, but on-going research within many of these techniques will undoubtedly lead to the development of improved prediction approaches in the future.

## Data Availability

Data from Craig, unpublished is available upon request from the authors.
